# Geographical targeting of active case finding for tuberculosis in Pakistan using hotspots identified by artificial intelligence software (SPOT-TB): study protocol for a pragmatic stepped wedge cluster randomised control trial

**DOI:** 10.1136/bmjresp-2023-002079

**Published:** 2024-07-11

**Authors:** Syed Mohammad Asad Zaidi, Amna Mahfooz, Abdullah Latif, Nainan Nawaz, Razia Fatima, Fazal Ur Rehman, Tahira Ezra Reza, Faran Emmanuel

**Affiliations:** 1 WHO Centre for Tuberculosis Research and Innovation, Institute for Global Health, University College London, London, UK; 2 Center for Global Public Health, Islamabad, Pakistan; 3 Mercy Corps, Islamabad, Pakistan; 4 Ministry of National Health Services Regulation and Coordination, Islamabad, Pakistan; 5 University of Manitoba, Winnipeg, Manitoba, Canada

**Keywords:** Tuberculosis, Respiratory Infection

## Abstract

**Introduction:**

Pakistan has significantly strengthened its capacity for active case finding (ACF) for tuberculosis (TB) that is being implemented at scale in the country. However, yields of ACF have been lower than expected, raising concerns on its effectiveness in the programmatic setting. Distribution of TB in communities is likely to be spatially heterogeneous and targeting of ACF in areas with higher TB prevalence may help improve yields. The primary aim of SPOT-TB is to investigate whether a policy change to use a geographically targeted approach towards ACF supported by an artificial intelligence (AI) software, MATCH-AI, can improve yields in Pakistan.

**Methods and analysis:**

SPOT-TB will use a pragmatic, stepped wedge cluster randomised design. A total of 30 mobile X-ray units and their field teams will be randomised to receive the intervention. Site selection for ACF in the intervention areas will be guided primarily through the use of MATCH-AI software that models subdistrict TB prevalence and identifies potential disease hotspots. Control areas will use existing approaches towards site selection that are based on staff knowledge, experience and analysis of historical data. The primary outcome measure is the difference in bacteriologically confirmed incident TB detected in the intervention relative to control areas. All remaining ACF-related procedures and algorithms will remain unaffected by this trial.

**Ethics and dissemination:**

Ethical approval has been obtained from the Health Services Academy, Islamabad, Pakistan (7–82/IERC-HSA/2022–52) and from the Common Management Unit for TB, HIV and Malaria, Ministry of Health Services, Regulation and Coordination, Islamabad, Pakistan (26-IRB-CMU-2023). Findings from this study will be disseminated through publications in peer-reviewed journals and stakeholder meetings in Pakistan with the implementing partners and public-sector officials. Findings will also be presented at local and international medical and public health conferences.

**Trial registration number:**

NCT06017843.

**WHAT IS ALREADY KNOWN ON THIS TOPIC**

- Active case finding (ACF) is a potential strategy to increase case detection of tuberculosis (TB) by systematic screening of communities outside of healthcare facilities. Recent evidence shows that ACF reduces population-level TB incidence and prevalence through early detection of TB. However, ACF interventions are resource-intensive and strategies to increase yields of TB detected need to be explored. Tuberculosis epidemics are characterised by spatial heterogeneity rather than geographically uniform risk, suggesting that targeting of ACF in areas with higher TB prevalence may help improve yields. However, there has been no prospective study that has evaluated a targeted approach towards ACF in a high TB burden setting.

**WHAT THIS STUDY ADDS**

- SPOT-TB is a pragmatic, stepped wedge cluster randomised trial that will evaluate whether a targeted approach towards ACF, supported by artificial intelligence software (MATCH-AI), can increase yields of TB detected. The study is being conducted on a national scale in Pakistan and is embedded within the routine operations of the implementing partners of the National TB Programme. The study will provide empirical evidence for the potential benefit of targeting of ACF interventions in a real-world setting, within a high-burden TB country.

**HOW THIS STUDY MIGHT AFFECT RESEARCH, PRACTICE OR POLICY**

- The study will help inform whether a targeted approach for ACF in high TB burden countries is effective and feasible. The study may inform national and global guidelines and policy related to ACF in high-burden countries. The study evaluates an artificial intelligence software that can be readily deployed in other settings to support global TB elimination efforts, should it prove to be effective.

## Introduction

 Globally, an estimated 10.6 million individuals fell ill with tuberculosis (TB) in 2021.^1^ Pakistan has the fifth highest TB burden in the world with an estimated incidence of 573 000 individuals with TB per year and 42 000 TB-related deaths occurring annually in the country.^2^ A key challenge in Pakistan’s efforts towards eliminating the disease is ensuring diagnosis and treatment of all individuals with TB. In 2020, of the estimated 573 000 individuals with TB, only 276 736 (48%) were diagnosed, highlighting a case-detection gap of more than 50%. Bridging this case-detection gap is a critical objective for the National TB Programme (NTP).^2^

Active case finding (ACF) is a potential strategy to increase case detection by systematic screening for undiagnosed TB outside of health facilities.^3 4^ Recent evidence indicates that in addition to increasing case detection in the short-term, ACF interventions, if sustained over a period of time, can also help reduce population-level TB incidence and prevalence.^5^,^7^ There is evidence to suggest that TB transmission may also take place subclinically, that is, in the absence of any symptoms by the individual.^8 9^ This suggests that focusing on passive diagnosis alone may be insufficient for TB elimination. In 2021, the WHO updated its systematic screening guidelines, supporting screening in the general population where TB prevalence is >0.5%.^10^

Pakistan has invested significantly in improving its capacity for active case finding including the use of mobile X-ray units with computer-aided detection (CAD) for screening and molecular diagnostics, such as Xpert MTB/RIF for diagnosis.^11^,^13^ These interventions are currently led by Mercy Corps-Pakistan, a private-sector implementing partner of the NTP and its subrecipients (SRs) and are supported by the Global Fund. While community-based ACF interventions (called ‘chest-camps’ or ‘camps’) are now being conducted at scale in Pakistan, they are resource and labour intensive. Concerns remain regarding the yields and cost-effectiveness of systematic screening in programmatic settings, particularly given competing priorities within TB programmes.^14^ A recent TB Joint Programme Review Mission (JPRM) conducted by the Pakistan government, WHO and other technical agencies identified low yields from ACF interventions and recommended the use of a geographically targeted approach for ACF in areas where TB prevalence is higher than the WHO threshold.^15^

Similar to other infectious diseases, TB epidemics may be characterised by areas with concentrated rather than spatially uniform risk.^16 17^ These are often described as disease ‘hotspots.’ A systematic review investigating methods used in spatial analysis of TB epidemiology identified heterogeneous spatial patterns in all included 168 studies.^18^ More recently, a study from Karachi, Pakistan also demonstrated significant spatial heterogeneity in detection of TB through community-based screening of 197 693 individuals over a 2-year period.^19^

In response to the JPRM recommendations, Mercy Corps, Pakistan has made a policy decision to use a geographically targeted approach towards ACF using MATCH-AI, an artificial intelligence (AI) software. This software uses a Bayesian modelling approach to predict areas of high TB prevalence and to identify potential hotspots to guide ACF site selection.^20^ While the software may potentially support with increasing yields from ACF, it has not been rigorously evaluated in the field setting. The MATCH-AI rollout therefore provides an opportunity to evaluate the impact of a geographically targeted approach towards ACF on TB detection through the use of AI software in a real-world, programmatic setting. In this paper, we describe a trial protocol that will assess the quantitative impact of the software within the existing ACF programme in Pakistan. A qualitative evaluation of the software’s acceptability and feasibility by the implementers is being investigated separately to this study.

## Methods and analysis

### Aim

The primary aim of the SPOT-TB trial is to compare the yield (number of individuals diagnosed with TB) during ACF camps using a site selection approach based on predictions generated via an artificial intelligence software (MATCH-AI) vs the conventional approach of camp site selection using local knowledge, experience and analysis of historical data by field staff. A secondary aim of this study is to strengthen capacity of the partnering organisations in routine programmatic data entry, management and analysis through implementation of electronic record systems.

### Trial design

SPOT-TB is a pragmatic stepped wedge cluster randomised control trial. In this type of study design, clusters successively switch from the control to the intervention arm in a randomised manner. Initially, all clusters are part of the control arm and by the conclusion of the trial, all clusters will be part of the intervention arm. SPOT-TB is designed to be highly pragmatic, with the study being fully embedded within routine, programmatic implementation of ACF by Mercy Corps and its SRs.

### Study setting

The study will be conducted in all four provinces of Pakistan within 72 districts (table 1). According to the last TB prevalence survey conducted in 2010–11, the overall prevalence of TB in Pakistan is per 398 per 100 000 population.^21^

**Table 1:** List of districts included in trial

Province	Districts
Balochistan	Chaghi, Duki, Gwadar, Jaffarabad, Kech, Khuzdar, Lasbela, Loralai, Nasirabad, Nushki, Panjgur, Pishin, Qilla Saifullah, Quetta, Sibi
Khyber Pakhtunkhwa	Bannu, Battagram, Charsadda, Chitral, Ghotki, Hangu, Haripur, Kohat, Lower Dir, Mansehra, Mardan, Nowshera, Peshawar, Upper Dir
Punjab	Attock, Bhakkar, Chiniot, Faisalabad, Ghotki, Gujranwala, Gujrat, Hafizabad, Jhang, Khanewal, Khushab, Layyah, Lodhran, Mandi Bahauddin, Multan, Okara, Pakpattan, Rawalpindi, Sahiwal, Sargodha, Toba Tek Singh, Vehari
Sindh	Badin, Dadu, Hyderabad, Jacobabad, Jamshoro, Karachi Central, Karachi East, Karachi Korangi, Karachi Malir, Karachi South, Karachi West, Karachi Kemari, Kashmore, Khairpur, Larkana, Matiari, Mirpur Khas, Naushahro Feroze, Qambar Shadadkot, Sanghar, Shaheed Benazirabad, Shikarpur, Sukkur, Tando Allahyar, Tando Muhammad Khan, Tharparkar, Umerkot

### Randomization

The unit of randomisation in this trial will be a mobile X-ray unit and its associated team members (van-team). Since van-teams are shared between districts, it is more appropriate to randomise the teams instead of individual districts. The number of districts covered by each van-team varies from 1 to 3 depending on the size and population of the districts. A total of 30 van-teams will be randomised to the intervention through a computer-generated list at the start of the trial. The randomisation process and schedule will be carried out independently by the research team. Van-teams will be masked to the intervention allocation and randomisation schedule until they switch to the intervention.

**Figure 1:**
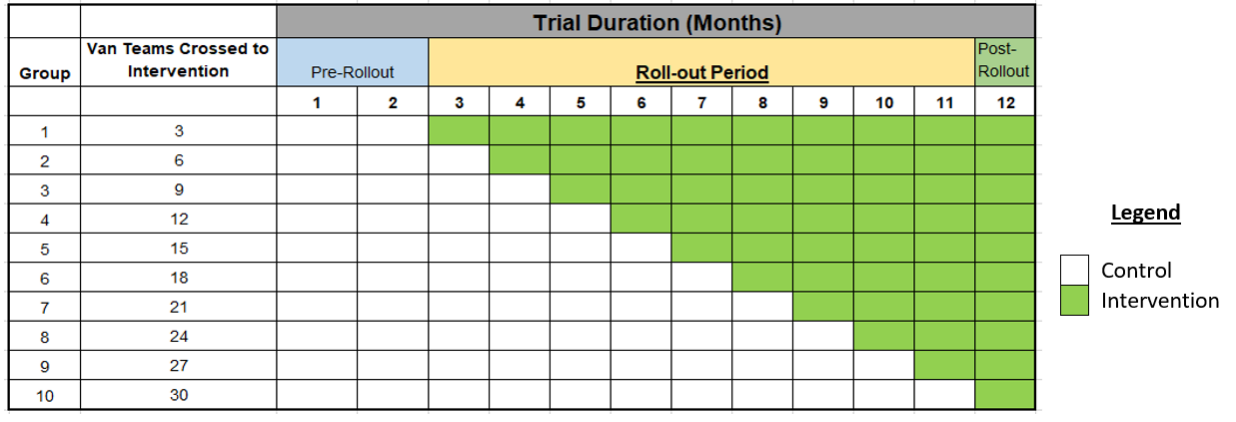
Stepped wedge implementation of geographically targeted active case finding for tuberculosis in Pakistan using artificial intelligence software.

The total proposed length of the study is 12 months (data collection period). At the start of the trial, no clusters will be using MATCH-AI. A 2-month pre-rollout period will be used to collect baseline control data and conduct pilot testing of the intervention. From month 3 onwards, three van-teams will be randomly selected to start using MATCH-AI for camp selection (figure 1). By the end of the trial, all 30 teams will be using MATCH-AI for camp planning.

### Study intervention

#### Overview of MATCH-AI

The primary intervention in this study is the roll-out of MATCH-AI, an artificial intelligence software that models subdistrict TB prevalence. This software will be used to guide site selection of ACF camps. The software has been developed by the KIT Royal Tropical Institute in the Netherlands and Epcon, a commercial software firm.^22^ The MATCH-AI tool uses a Bayesian modelling approach to predict TB prevalence to a resolution of 10 000 population polygons. The model integrates data from a range of sources including historical TB facility notification data, previous ACF data as well as contextual factors such as demographics, income, population density, health indicators such as vaccination coverage and climate-related variables to predict localised TB prevalence. The software features a dashboard that allows for field staff to visualise maps that overlay TB prevalence and to help identify camp locations. The platform is self-learning and incrementally improves the prediction as further data is entered into the system.

In the intervention arm, camps will be conducted primarily in locations guided by MATCH-AI. Van-teams will be provided with a list of the highest predicted TB prevalence polygons (using street addresses and GPS coordinates) in each district as predicted by MATCH-AI. Van-teams will themselves not use the software interface. Instead, locations will be communicated by the Mercy Corps head office (via monthly emails) and van-teams will be instructed to move sequentially through this list to conduct ACF activities, starting with locations with the highest predicted prevalence. Once van-teams have switched over to the intervention group, they will continue to use MATCH-AI to guide their decision-making through to the end of the trial. Teams that are scheduled to receive the intervention will be provided with the list of locations 15 days prior to the start of the month, allowing sufficient time for them to transition to the intervention and to plan their community engagement activities. Van-teams will have the option to repeat camps at the same locations should they provide high yields. However, they will be encouraged to also screen additional locations within the list. A minimum (unspecified) number of camps will be allowed independent of the MATCH-AI predictions due to any prior commitments of van-teams with local communities or due to logistical constraints in reaching AI-directed sites.

#### Current standard of care

In the control arm, field teams will continue to use existing approaches towards camp site selection. While not using software to guide decision-making, field staff may include targeting in their approach towards site selection. This is based on local knowledge, field experience from previous camps, analysis of historical data in paper-based TB registries and consultations with local stakeholders such as district TB coordinators and health officers. Camp plans are approved by the head office each month after reviewing recommendations of the district teams. Camp sites are often focused in slum dwellings and occasionally target key populations such as injection drug users. In addition, camps are often conducted in collaboration with local non-profits and private healthcare facilities such as general practitioner (GP) clinics. However, the current approach of camp site selection does not systematically use geographical targeting or quantitative methods to identify TB hotspots.

### Eligibility criteria

A total of 72 districts of Pakistan that have mobile X-ray and Xpert testing facilities will be enrolled into the study (table 1). Districts where mobile X-ray units and Xpert machines are unavailable will be excluded. This will ensure that camp procedures and algorithms are uniform across the intervention and control arms.

All individuals presenting to the camp sites will be eligible to participate for screening, including those with previous history of TB disease as per routine ACF guidelines. Children <12 years of age and pregnant women will be excluded from screening as per NTP guidelines that restrict these subgroups from inclusion in mass-screening interventions in the community setting.

### Participant recruitment and screening algorithm

**Figure 2:**
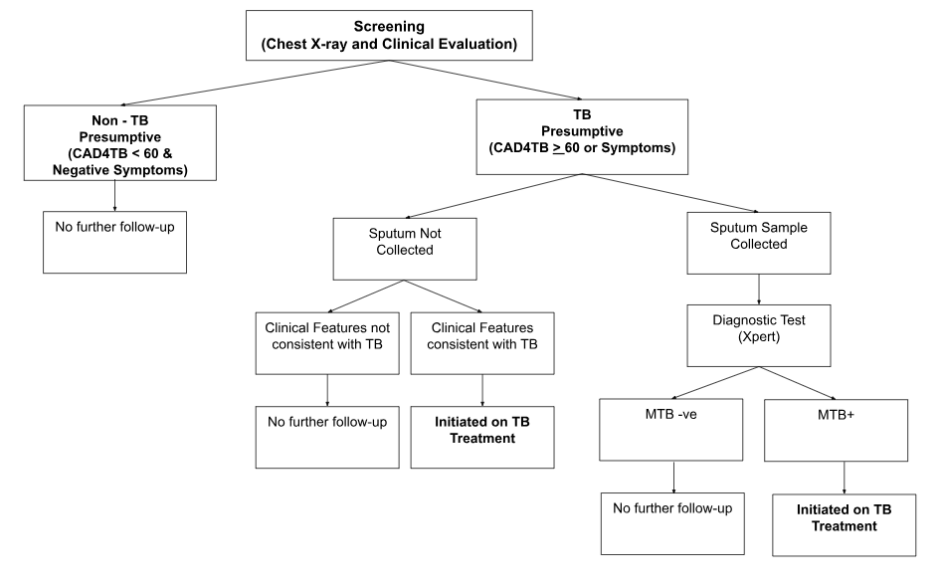
Screening and diagnostic algorithm during active case-finding camps in Pakistan. This algorithm will remain unaffected by the trial and will be uniformly used in both intervention and control arms.

Participants will be drawn from ACF camps conducted by Mercy Corps. Camps is a commonly used term within the TB programme to describe a single day’s ACF activity. Camps are conducted in public places such as street corners, parks, mosques or busy market places where neighbourhood residents or visitors can easily participate in screening. These typically involve setting up of a screening site with tarpaulin coverings for shade, prominent banners, tables and chairs. With increasing use of mobile X-ray units, less setup is required and vans are parked at the intended locations and people from the surrounding community are encouraged to participate using loudspeaker announcements and outreach by fieldworkers. Existing procedures for community mobilisation, screening, diagnosis and treatment will be used in both arms and will remain unaffected by this trial (figure 2). Once camp locations are finalised, meetings with ‘area notables,’ such as community elders and local politicians are conducted a few days prior to the activity. The goal of these meetings is to use the social networks and influence of notable individuals to increase community participation in the upcoming camp. Banners and posters are placed at prominent locations, pamphlets are distributed and announcements are made in mosques. Number of participants at camps on average are 100 (±56) individuals during a single day’s activity.

On the day of the camp, the mobile X-ray unit is brought to or near the intended camp location. All Individuals are screened for TB using chest X-rays that are interpreted by computer-aided detection software (CAD4TB). Individuals with the CAD4TB score of >60 are considered to be TB presumptives. Individuals with scores <60 are directed towards a medical officer for further evaluation. The medical officer may choose to request sputum investigation from these individuals on the basis of clinical examination. All TB presumptives (individuals with CAD4TB >60 or those identified by the medical officer) are encouraged to provide a sputum sample specimen onsite. Individuals who are not able to expectorate a sputum sample are provided containers with instructions on producing a morning specimen. No further workup is conducted for individuals that are not considered to be presumptive for TB. Collected sputum samples are tested using Xpert onsite in the mobile X-ray unit or transported to a nearby designated public or private-sector laboratory with Xpert testing facility. Individuals testing positive on Xpert or those diagnosed empirically by the medical officer are initiated on treatment. Individuals with a negative Xpert may choose to visit a physician at a static medical facility or clinic but are not actively followed up by the programme.

### Consent

Verbal consent will be sought from individuals undergoing screening at camps. Verbal assent will be sought from parents or guardians of children <18 years of age. The existing participant information sheet used by implementers during screening has been adapted for the purposes of the trial (online supplemental file 1).

### Data handling and record keeping

This pragmatic trial will use routine data collection processes to evaluate the intervention. At the conclusion of each camp, field staff led by a District Field Supervisor (DFS) aggregate data related to key indicators (box 1). These indicators have been adapted for the purposes of the evaluation. To validate whether camps took place at the indented locations predicted by MATCH-AI, the study team will independently monitor existing geo-tracking system of the mobile X-ray vans. Aggregate data from each camp will be stored on an encrypted and secure project server. All data used in this trial is aggregate and fully anonymous. The trial makes no use of personal data.

Box 1 **Summary indicators recorded at conclusion of each active case-finding camp**

**CampOverview**

Name of the district
Area notable meeting conducted
Total visitors at the camp (male)
Total visitors at the camp (female)
Camp venue

**Patient Indicators**

Total visitors
Number of chest X-rays conducted
Number of chest X-rays with CAD >60
No. of sputum tested through Xpert
No. of sputum samples sent to static laboratory for microscopy
Total individual registered with B+TB
Total individuals registered with All-Forms TB
Total individuals with rifampicin resistance detected

**Location and Accessibility**

Nearest health facility (distance in km)
Type of health facility (BHU, RHC, THQ, DHQ, GP)
Camp location (school, mosque, park, etc)
Camp accessibility (within neighbourhood, walking distance, not walking distance, distant)
Road quality (metaled, dirt track, mixed, no road)

**Camp Details**

Camp site selection method (MATCH-AI, district team, district TB official, other)
Previous camps in location
Weather on camp day
Local events during camp day

**Staff and Facilities**

Screening method (symptoms, X-ray, X-ray with CAD)
Diagnostic method (Xpert, AFB smear)
Diagnostic testing site (van, laboratory)
Transportation method (District Field Supervisor, rider)
Educational material distributed
List of staff at camp
Qualification of physician
Physician experience

**Community Mobilisation**

Use of public announcements, LHWs, volunteers, school children, local doctors
Involvement of mosque imam, area notable, commissioner, local leader, village councillor

AFB:, Aacid Ffast Bbacilli,; BHU:, Bbasic Hhealth Uunit,; CAD:, Ccomputer- Aaided Ddetection,; DHQ:, District Headquarter Hospital; GP:, Ggeneral Ppractitioner; LHW:, Llady Hhealth Wworker,; RHC:, Rural Health CenterCentre,; THQ:, Tehsil Headquarter Hospital

Initially, the DFS will record camp data manually and enter data on to the project server using *KoboToolBox* software. An extensive staff training programme has been developed to support routine data collection and to strengthen research capacity within the implementing partners during the trial rollout. This includes the concurrent implementation of an electronic data collection and reporting tool using tablet devices that will replace paper-based forms and registries currently in use. The new data collection tools will include validation checks to prevent errors in data entry. Data officers from the project team will conduct independent reviews of the quality of data entry and will conduct field audits, including cross-verification of reported data from TB registers. Aggregate data used in the final analysis will be made publicly available and stored in an online repository for long-term archiving.

### Outcome measures

The primary outcome measure in this study will be Camp Positivity Yield, defined as counts of bacteriologically confirmed TB (B+) diagnosed in each camp (table 2). Secondary outcome measures include Camp Positivity Rate, defined as, B+/camp participants, Camp All-Forms Yield, defined as counts of All-Forms TB (AF-TB) diagnosed in each camp and Camp All-Forms TB Rate, defined as AF-TB diagnosed/camp participants.

**Table 2:** SPOT TB Outcome measures

Outcome	Measures	Time of measurement
Primary outcome		
Camp positivity yield	Counts of bacteriologically confirmed TB (B+) diagnosed in each camp	1–2 days after each camp
Secondary outcomes		
Camp positivity rate	Bacteriologically confirmed TB (B+)/camp participants	1–2 days after each camp
Camp All-Forms TB yield	Counts of All-Forms TB (AF-TB) diagnosed in each camp	1–2 days after each camp
Camp All-Forms TB rate	All-Forms TB (AF-TB) diagnosed/camp participants	1–2 days after each camp

TBtuberculosis

### Sample size

Since this intervention is being rolled out programmatically, the sample size will not be capped. Using camp data from 2022, we calculated a mean of 0.88 B+ diagnosed per camp with an over dispersion of 1.34. We calculated intra-cluster correlation (ICC) of 0.12 using one-way analysis of variance (ANOVA) of B+ and van-clusters. We subsequently used ‘*The Shiny CRT X Calculator: Power and Sample size for Cluster Randomised Trials Test*’ to conduct the sample-size calculation for stepped-wedge trial using a cross-sectional sampling structure assuming an exchangeable correlation structure and significance level of 0.05.^23^ We anticipate each van to conduct approximately 10–13 camps per month (cluster-size), accounting for exclusion of camps in districts without Xpert testing and cancellations of camps due to external factors. With 10 steps and three vans switching per cluster, the trial is powered at 80% to detect a 20% increase in B+ in the intervention arm assuming an average of 10 camps per month or 3300 total camps over the study period. A power of 90% is achieved if 13 camps per month are undertaken during the study period or 4290 total camps over the study period.

### Statistical analysis plan

Frequency statistics will be used to describe the baseline characteristics of camps and the participants (box 1). These will be compared between the intervention and control arms to assess the appropriateness of the randomisation process and tested for statistical significance using parametric and non-parametric tests as appropriate.

The primary analysis will use a Poisson generalised liner mixed regression using the number of bacteriologically positive TB diagnosed per camp as the outcome variable. This may be modified to a mixed negative binomial regression, depending on the dispersion of data and model goodness of fit. To account for clustering of outcomes within the randomisation unit (van-teams), they will be added as a random effect in the model. Due to secular trends in the incidence of TB, a fixed effect for time (monthly) will be added into the model, based on the trend in B+ reported. TB notifications are typically higher in the summer months in Pakistan; however, it is unclear whether this is due to health-seeking behaviour or disease pathophysiology. Camps will be categorised into intervention or control based on their exposure to the MATCH-AI intervention and added as a binary explanatory variable into the model to calculate the incidence rate ratio between the two arms. The primary analysis will be intention-to-treat where all camps will be considered to have switched to the intervention group after their assignment, regardless of whether those camps were conducted using MATCH-AI. In the per-protocol analysis, only camps conducted at the location identified by the MATCH-AI software will be included. Camps conducted within a 2-km radius of the intended camp site (validated using GPS coordinates) will be included in the per-protocol analysis. A subgroup analysis will be carried out by stratifying the effect of the intervention over regional prevalence estimates of TB (high, medium, low) identified through a retrospective analysis of mobile X-ray-based camps in Pakistan conducted prior to the start of the trial.

### Trial oversight

A Trial Oversight Committee will provide day-to-day decisions and oversight of the project. It will include the principal investigator (PI), co-PIs and the National Coordinator (NC). The committee will provide technical inputs and back stopping for all technical and operational issues related to the study. It will be responsible for maintaining coordination among all partners and field teams, planning and executing all field activities and ensuring logistics and communication. The trial oversight committee will report to a Technical Advisory Group (TAG) comprising of project investigators, representatives from Mercy Corps, field implementing organisations and technical staff of NTP. The TAG will provide high-level guidance to the project, will review research strategies, disseminate the results and follow-up on research-related policies. A Data Monitoring Committee (DMC), chaired by the project NC, will be responsible for reviewing field data collection and monitoring. The DMC will work closely with field staff to ensure timely and accurate data collection and reporting and will conduct monthly data audits in the field. The DMC will share validated datasets periodically with the investigators, including for interim and final analysis.

### Role of software developers

The MATCH-AI software, the underlying prediction model and the dashboard interface have been developed independently of the study team. The developers have had no involvement in the design and conduct of this external evaluation.

### Ethics and dissemination

Ethical approval for this trial has been provided by the Institutional Review Board (IRB) of the Health Services Academy, Islamabad, Pakistan (IRB Number: 7–82/IERC-HSA/2022–52). Ethical approval has also been obtained from the Common Management Unit for TB, HIV and Malaria, where the National TB Programme is based at the Ministry of Health Services, Regulation and Coordination, Islamabad, Pakistan (IRB Number: 26-IRB-CMU-2023). Since this trial evaluates a policy change in the selection of camp sites, no specific consent procedures related to the study have been developed. There are minimal risks to participants directly from the study. All existing consent procedures for screening during ACF used by the implementing partners as part of the routine programmatic activities will remain unaffected by this trial.

The findings from this study will be disseminated through publications in peer-reviewed journals. They will also be disseminated through stakeholder meetings in Pakistan with the implementing partners and public-sector officials. Results will be presented at international conferences and symposia to support wider dissemination within the scientific and global health community.

### Patient and public involvement

Patients or members of the public were not involved in the design of the study. They will also not be involved in the conduct or reporting of the study. We aim to include members of the public and affected communities during dissemination of the study results. Their input will be sought to develop appropriate public messaging for ACF in potential hotspot areas, should the tool prove to be effective. This process will eventually support the development of a community advisory board to help inform future applied research on ACF in Pakistan.

## Discussion

To our knowledge, SPOT-TB is the first prospective trial investigating the use of a targeted approach towards ACF in a high TB burden country. A previous systematic review of spatially targeted screening interventions for TB by Cudahy *et al* identified only three studies addressing this research question, all of which were conducted within the USA, a low TB burden country.^24^ The known spatial heterogeneity in TB epidemiology as well as evidence from modelling studies provides a strong *theoretical* basis for targeted screening approaches. This study will fill an important gap in the *empirical* evidence for geographically targeted ACF and will assess its effectiveness in a high-prevalence setting. If the intervention proves effective, this study may influence the wider debate on ACF as a strategy for TB elimination and influence its implementation in high TB burden countries.

A number of challenges may have prevented researchers from addressing this question in the past. These consist of costs associated with conducting ACF trials and achieving sufficient power in a prospective study design, availability of mass screening and diagnostics for TB, deploying data systems at scale and practicalities of identifying and screening in high-prevalence areas. A key feature of this trial is to leverage several recent technical, operational and policy developments that provided an opportunity to conduct this trial in Pakistan. These include growing recognition of early TB states, in particular subclinical – infectious state on *Mycobacterium tuberculosis* transmission, development of CAD software for rapid community screening, investments in mobile X-ray units for ACF and rapid molecular testing at the district level in Pakistan.^1225^,^28^ These developments together with advances in AI that can provide precise estimates of TB prevalence have enabled localised targeting of ACF interventions. Most significantly, the trial has strong institutional and policy support from implementing partners and the NTP that are committed to expanding ACF but are cognizant of the resource implications. Exploring innovations that have been proven to be effective in the Pakistani setting and can be easily incorporated as part of routine programme planning and monitoring is an important priority for the NTP.

A strength of this trial is that it is designed to be highly pragmatic and is fully embedded within routine activities of the programme.^29 30^ All existing operational procedures for conducting ACF camps will remain unaffected including services delivered to participants visiting camps. This pragmatic approach has not only made it feasible to conduct such a trial but it will also help ensure that the findings from this study will have relevant policy implications for program managers in Pakistan and elsewhere. Randomisation was considered appropriate at the van-team level as it allows measurement of the effectiveness of the policy change at the intended users of the software. Randomisation of van-teams instead of individual camps has the added benefit of preventing potential overlap of locations selected by both the software and field staff. A qualitative evaluation of the intervention’s acceptability and feasibility is being concurrently investigated by the researchers with a focus on how use of the software affects workflows and processes of implementers at the district level.

A stepped wedge design was chosen to facilitate the rollout of the intervention in a sequential approach. This will allow the implementers to gain experience in the rollout of the software, identify logistical challenges early and help incrementally improve the implementation. Given the scale of ACF programme in Pakistan, it would have been practically challenging to simultaneously implement the tool across half of the districts in a parallel cluster randomised design. A stepped wedge design will offer a more methodologically robust evaluation relative to a before-and-after design or other non-randomised study designs while also facilitating the project implementation.^31^ We hypothesise that the intervention will help increase TB case detection and reduce community transmission of TB. The stepped wedge design therefore also prevents ethical concerns from withholding of a potentially beneficial intervention in the control areas. Finally, a stepped wedge design will help increase statistical power of the trial relative to other study designs given the high ICC observed in TB detection in the baseline data. This is due to increased precision resulting from clusters initially serving as their own controls.^32^

A limitation of the trial design is that field staff will not be masked to the intervention status once they have switched over from the control group. Another limitation inherent in the design is that van-teams will conduct a certain proportion of camps in locations independent of the MATCH-AI recommendation. This reflects a pragmatic compromise whereby teams will be able to retain some autonomy in the selection of a camp due to commitments with local partners such as private GPs, non-governmental organisations and community groups. These camps have been retained in the primary analysis to make the results more externally generalisable as programmes would likely encounter such practical considerations. The per-protocol analysis will exclude these camps and will provide a direct estimate of the intervention effect. We anticipate the trial implementation to be affected by logistical challenges common in programmatic implementation such as sputum collection and testing, data reporting accuracy and external disruptions. A randomised evaluation will help ensure that these constraints do not bias results towards either the intervention or control arms whereas appropriate statistical methods and secondary analyses will adjust for other confounders. A potential limitation of the stepped wedge design is that secular trends in TB incidence may affect outcomes between intervention and control groups. This will be adjusted for using a fixed effect for time (monthly) in the regression models. An extensive staff training and capacity-building programme along with monitoring will help minimise data collection and reporting errors. An electronic data-collection tool will be concurrently rolled out to support long-term capacity for research within the implementing partners in Pakistan.

While this trial is evaluating the use of a targeted approach towards ACF, it is reliant on a commercial software to guide decision-making. The results of the trial will therefore need to be interpreted with caution in the overall context of geographically targeted screening of TB. The output of the software is dependent on the priors and distributions used in the Bayesian modelling for estimating TB prevalence. It is likely that an alternative platforms or models will lead to different location recommendations. Since the trial results are contingent on the effectiveness of the software, they may not necessarily be generalisable to all geographically targeted approaches towards ACF. Further studies using alternative hotspot identification methods, as well as in other high TB burden settings, particularly in countries with a high HIV prevalence, should be considered to build further evidence for targeting of ACF. The trial has a relatively short timeline (12 months) and it is unclear how the software will perform if repeated screening at the same locations leads to epidemiological changes in the target population. While the model includes yields from ongoing ACF data within its predictions, assessing the longer-term performance of the software is beyond the scope of this evaluation, which is restricted to addressing the immediate programmatic priority, that is, low yields from ACF. A cost-effectiveness analysis was also beyond the scope of this evaluation and may be considered to further inform policy related to geographical targeting and use of the software.

SPOT-TB will evaluate an innovative, artificial intelligence based approach towards geographically targeted active case finding for TB through a pragmatic stepped wedge cluster randomised trial. If the intervention proves to be successful, the study can make positive recommendation to TB programmes in Pakistan and other high TB burden countries regarding the use of this software to guide TB elimination efforts.

## supplementary material

10.1136/bmjresp-2023-002079 online supplemental file 1

## Data Availability

Data are available in a public, open access repository.
