# Supplementary material for: Geographical targeting of active case finding for tuberculosis in Pakistan using hotspots identified by artificial intelligence software (SPOT-TB): study protocol for a pragmatic stepped wedge cluster randomised control trial
Source: BMJ Open Respir Res. 2024 Jul 11;11(1):e002079. doi: 10.1136/bmjresp-2023-002079 (PMC11243128; doi:10.1136/bmjresp-2023-002079)
Supplement: online supplemental file 1 [file bmjresp-11-1-s001.pdf]

# Information sheet

*Instructions for chest camp in-charge or District Field Supervisor (DFS):*

- *Use this form for participants who are over 18 years of age.*
- *The DFS or camp in-charge should read out the consent form to all attending participants at the time the participants receive their OPD token slip for the chest camp.*
- *The DFS or camp in-charge would mark the consent and sign it on behalf of the participant as the consent is obtained.*

An evaluation study is being conducted by the Centre for Global Public Health Pakistan in partnership with the Mercy Corps to assess the effectiveness of Artificial Intelligence to identify hotspots for TB cases compared to the conventional method of selecting the hotspots. This evaluation is being done in all chest camps organized by SR organizations across all 90 districts of Pakistan. There will be no separate interview for this, you will be registered and tested as per the usual practice, but your information other than your name will be used in the evaluation study.

The results of this study will lead us to identify the method which is better in identification of hotspots for finding missing TB cases which in turn will decrease the overall burden of TB from the country. We will also develop a report, but YOU WILL NOT BE NAMED OR IDENTIFIED IN ANY WAY in this report because WE DO NOT NEED TO KNOW YOUR NAME or any other relevant information which can be used to identify you. If you do not want us to use your information for the study, you are completely allowed to refrain us from using your information. By refusing to use your information for the evaluation study will not affect provision of any services within this camp. All diagnosis and treatment will be provided to you as per routine.

There is no coercion of any type for you to participate in this evaluation. If you have any questions about your rights as a subject participating in a research survey, or if you wish to discuss your participation in the study, you can contact Ms. Amna Mahfooz, Co-Investigator at CGPH-Pakistan on 051-8357603.

As this study is confidential, we do not want you to sign anything. If you agree to participate, I will mark yes on the consent form indicating your consent for participation.

| | | | | | |
|---|---|---|---|---|---|
| **DO YOU AGREE TO PARTICIPATE?** | **Yes** | ☐ | **No** | ☐ | **(Clarify/Discuss)** |

*if yes, sign and date below to indicate that informed consent was given by the participant)*

Name and signature of DSF/camp in-charge: ________________________________________

Date: _________________________________
